# Beyond the Group Mean: Individual Response Heterogeneity in Social Appearance Anxiety, Anxiety and Interpersonal Functioning Following Aesthetic Surgery—A Prospective Controlled Study

**DOI:** 10.3390/jcm15207982

**Published:** 2026-10-15

**Authors:** Özcan Öcük, Şengül Kocamer Şahin

**Affiliations:** 1 Department of Plastic, Reconstructive and Aesthetic Surgery, Gaziantep Hatem Hospital, 27220, Gaziantep, Türkiye; ozcanocuk@gmail.com; 2 Department of Psychiatry, Adana Medical Faculty, Adana City Research and Training Hospital, University of Health Sciences, 01230, Adana, Türkiye

**Keywords:** aesthetic surgery, individual response heterogeneity, reliable change, social appearance anxiety, interpersonal relationships, equivalence testing

## Abstract

**Background/Objectives**: The psychological consequences of elective aesthetic surgery are usually evaluated by comparing group means, an approach that is insensitive to change that differs in direction across individuals. This prospective controlled study examined both group-level and individual-level psychological change following aesthetic surgery. **Methods**: Forty adults undergoing elective aesthetic procedures and thirty-nine comparison participants completed the Social Appearance Anxiety Scale (SAAS), an anxiety inventory, and the Interpersonal Relationships Dimensions Scale (IRDS) at baseline and three months later. Analyses comprised mixed-design repeated-measures ANOVA, linear mixed-effects models, Aligned Rank Transform ANOVA, two one-sided equivalence tests, Bayes factors, variance-of-change comparisons, three-month stability coefficients, and Reliable Change Index classification. **Results**: At baseline the surgical group reported higher anxiety (d = 0.61) and a distinct interpersonal profile, with markedly higher approval dependence (d = 1.64) and lower empathy (d = −1.46) and emotional awareness (d = −0.58). No time × group interaction was significant under any analytic framework (all FDR-adjusted *p* > 0.67). Equivalence testing established that differential effects larger than d = 0.64 could be excluded for five of seven outcomes, while Bayes factors provided only moderate support for the null (BF_01_ = 2.05–4.28). Individual-level analyses told a different story. The variance of change was 2.3 to 51.8 times larger in the surgical group (all FDR *p* < 0.05), three-month stability was substantially lower in the surgical group on six of seven outcomes, and 72.5% of surgical patients showed reliable change on at least one outcome compared with 12.8% of controls (*p* < 0.001), with reliable improvement (25.0%) and reliable deterioration (30.0%) occurring in broadly comparable proportions. **Conclusions**: The surgical group showed substantially greater variability in individual psychological trajectories than the comparison group despite the absence of differential group-mean change. These exploratory findings are compatible with possible individual response heterogeneity but should not be interpreted as evidence that aesthetic surgery itself caused psychological improvement or deterioration. Future research should examine whether preoperative characteristics can help identify patients who may be more likely to experience unfavorable psychological trajectories.

## 1. Introduction

In modern societies, physical appearance plays an important role in social acceptance, self-concept, interpersonal relationships and psychological health. Global interest in aesthetic surgery has grown alongside social comparison processes, shifting beauty standards and increased exposure to appearance-focused media [1,2]. Social Comparison Theory holds that people evaluate their appearance by contrasting it with that of others, and upward comparisons against idealised norms may increase interest in cosmetic procedures [1]. Although aesthetic surgery is undertaken primarily for appearance-related reasons, its psychological antecedents and consequences are complex and heterogeneous [3,4].

Contemporary social and digital environments may intensify appearance-based comparison by exposing individuals to idealised and digitally modified images. Such exposure may promote internalisation of appearance ideals, body dissatisfaction, self-awareness, and perceived social pressure. Nerini et al. linked social influences and public self-awareness with body dissatisfaction and acceptance of cosmetic surgery, while Mironica et al. similarly highlighted associations between social media exposure, body-image concerns, and consideration of cosmetic procedures. These mechanisms may also be relevant to social appearance anxiety and approval dependence, as both involve sensitivity to perceived evaluation by others [5,6].

Individuals seeking aesthetic surgery frequently report dissatisfaction with particular physical attributes, concern about being judged by others, and a wish to improve self-esteem. Social appearance anxiety is conceptually related to, but narrower than, general social anxiety. Whereas social anxiety concerns fear of negative evaluation across a broad range of interpersonal situations, social appearance anxiety specifically reflects concern about being judged on the basis of overall physical appearance. The SAAS was therefore selected because it directly captures the appearance-related evaluative concerns most relevant to individuals considering aesthetic procedures. At the same time, both constructs share a social-evaluative component, and their measurement requires attention to the possibility that self-report instruments may function differently across groups. More broadly, psychometric work on social-anxiety measures has emphasized the importance of establishing measurement invariance before interpreting between-group differences as substantive psychological differences, as illustrated by Baroni et al. in their evaluation of the Liebowitz Social Anxiety Scale–Self-Report [7]. Social appearance anxiety—the fear of being negatively evaluated on the basis of one’s overall physical appearance—has attracted particular attention [8,9,10]. Elevated social appearance anxiety has been associated with reduced quality of life, avoidance behaviour and impaired social functioning [11,12].

Aesthetic surgery may also influence broader emotional processes. Preoperative anxiety can arise both from anticipating the outcome and from dissatisfaction with appearance [13], and whether surgery produces durable reductions in anxiety remains contested [14]. Some studies report postoperative gains in body satisfaction and self-esteem, whereas others find no psychological change or only short-lived effects [15,16].

Interpersonal functioning is a further domain that surgery might affect. Perceived attractiveness, social confidence and sensitivity to approval or rejection all shape interpersonal dynamics [17]. Approval dependence, empathy, trust in others and emotional awareness are central dimensions of interpersonal functioning [18], and these may improve alongside increased body satisfaction or remain stable as relatively enduring personality-related characteristics [19].

### 1.1. Why Group Means May Be the Wrong Lens

A systematic review of the psychosocial outcomes of cosmetic surgery concluded that the evidence base remains largely uninformative, with substantial heterogeneity in design, comparison groups, measures and analysis across the prospective controlled studies conducted to date [20]. Almost without exception, those studies evaluate outcome by comparing group means over time. This is an appropriate way to estimate an average effect, but it is structurally blind to a pattern that clinical experience in aesthetic surgery suggests is likely: change that is substantial in individual patients yet opposite in direction across them.

There are several reasons to expect exactly that pattern here. Narrative and systematic reviews converge on the observation that a subgroup of aesthetic surgery patients—particularly those with body dysmorphic disorder, unrealistic expectations or pre-existing mood and anxiety disorders—show no improvement or an actual decline in psychological well-being after surgery, while others report meaningful benefit [14,21,22]. If both trajectories are present in the same cohort in comparable numbers, then the mean change will be approximately zero and a repeated-measures analysis will return a null result, irrespective of how much psychological movement has actually occurred.

Methodological work outside psychology has formalised this problem. Where individuals differ in their response to an intervention, the variance of pre-to-post change will tend to be larger in the intervention arm than in a comparator arm assessed over the same interval, because the comparator variance represents within-person random variation alone [23,24,25]. Comparing change variances therefore offers a preliminary probe for response heterogeneity that mean-based analyses cannot provide. Complementing this at the level of the individual patient, the Reliable Change Index [26] classifies each person’s change as exceeding, or not exceeding, what measurement error alone would produce, and remains the standard tool for reporting clinically meaningful change in outcome research [27].

A second limitation of the existing literature is inferential. Studies reporting no significant difference routinely describe their findings as showing that surgery has no psychological effect, which does not follow: a non-significant result is compatible both with the absence of an effect and with a study too small to detect one. Establishing the absence of an effect requires either an equivalence test against a pre-specified smallest effect size of interest [28,29] or a Bayesian quantification of the evidence favouring the null.

### 1.2. The Present Study

Accordingly, this study assessed social appearance anxiety, general anxiety and interpersonal relationship dimensions before elective aesthetic surgery and three months afterwards, relative to a comparison group. We predicted that surgical candidates would show higher baseline anxiety and appearance-related concern than controls and that group-mean change would not differ between the arms over the three-month follow-up. Exploratory post hoc analyses were subsequently conducted to examine whether individual psychological trajectories showed greater variability in the surgical group and whether reliable change occurred in both directions.

## 2. Method

### 2.1. Design

The study was conducted prospectively between January 2026 and April 2026. This was a prospective, observational, controlled pre–post study. Individuals undergoing aesthetic surgery were assessed before surgery and three months postoperatively. Changes in psychological variables were compared within the surgical group and between the surgical and control groups.

### 2.2. Participants

Participants in the surgical group were recruited from individuals scheduled to undergo elective aesthetic surgical procedures at Gaziantep Hatem Hospital. The procedures performed, in order of frequency, were abdominoplasty, breast surgery (augmentation or reduction), liposuction, Brazilian butt lift and rhinoplasty. The comparison group consisted of community volunteers recruited using a snowball sampling approach. No individual matching procedure was applied; rather, recruitment aimed to obtain a group broadly comparable to the surgical group in age and educational level. Individuals were not eligible for the comparison group if they had previously undergone aesthetic surgery, were currently planning an aesthetic procedure, or reported having seriously considered undergoing aesthetic surgery in the past. In addition, control participants were asked about current and past psychiatric treatment and psychiatric diagnoses; individuals with a current or previous diagnosed psychiatric disorder or a history of psychiatric treatment were excluded. Control participants were recruited independently of the psychological outcome data and were assessed at the same two time points as the surgical group. The same psychiatric exclusion criteria applied to the surgical group were also applied to the comparison group, including a history of severe psychiatric disorder and a diagnosis of body dysmorphic disorder.

Participants were eligible if they were between 18 and 50 years of age, provided written informed consent, and had sufficient cognitive capacity to complete self-report measures. Surgical participants additionally had to be scheduled for elective aesthetic surgery and medically eligible for preoperative assessment. Participants were excluded if they had a history of severe psychiatric disorder, were receiving psychiatric treatment or psychotropic medication, had a diagnosis of body dysmorphic disorder, were pregnant or breastfeeding, had a severe chronic medical condition, or were undergoing reconstructive or medically indicated rather than elective surgery.

**Figure 1:**
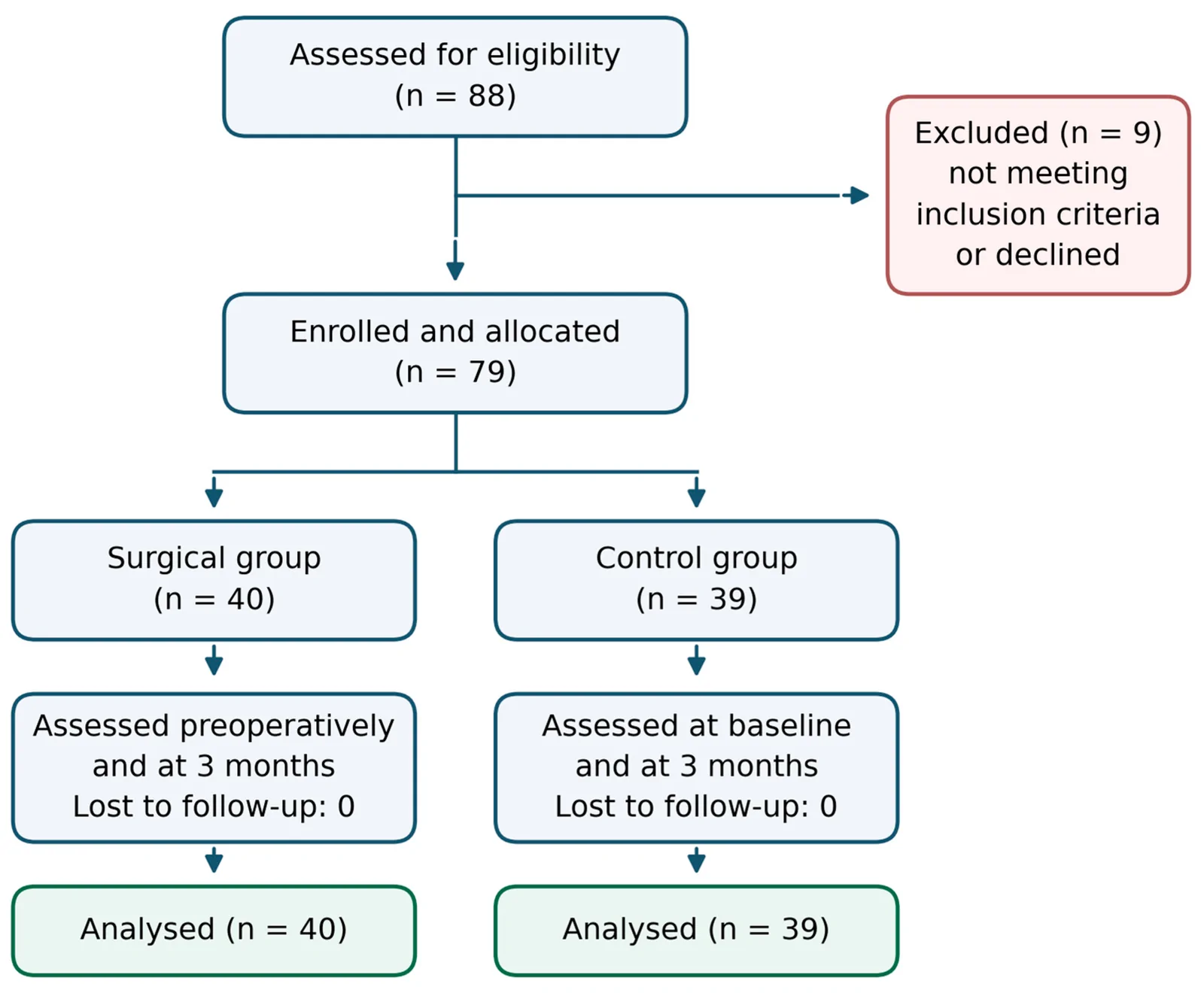
Participant flow. Of 88 individuals assessed for eligibility, nine were excluded in total, including one control participant whose response pattern showed invariant responding during data-quality screening. Seventy-nine participants were included in the final analyses: 40 in the surgical group and 39 in the comparison group.

Of 88 individuals assessed for eligibility, 79 were enrolled and all 79 completed both assessments: 40 in the surgical group and 39 in the control group (Figure 1). There was no loss to follow-up.

### 2.3. Measures

Social appearance anxiety was measured with the Social Appearance Anxiety Scale (Hart et al., 2008 [30], adapted into Turkish by Doğan (2010) [31]). The scale comprises 16 items rated from 1 to 5, with higher scores indicating greater social appearance anxiety; internal consistency in the present sample was acceptable (Cronbach’s α = 0.74).

Interpersonal functioning was measured with the Interpersonal Relationships Dimensions Scale [18], comprising 53 items across four subdimensions: Approval Dependence, Empathy, Trust in Others and Emotional Awareness. Approval Dependence reflects the extent to which individuals seek validation and acceptance from others and base their self-evaluation on external approval. Empathy assesses the ability to understand and respond to the feelings and perspectives of others. Trust in Others reflects the degree of confidence, openness, and perceived reliability experienced in interpersonal relationships. Emotional Awareness assesses the ability to recognize, understand, and express one’s own emotions. Higher scores indicate a stronger presence of the respective trait (Cronbach’s α = 0.86).

Anxiety levels were assessed using the Beck Anxiety Inventory, a 21-item self-report measure assessing anxiety severity over the previous week. Items are rated on a 4-point scale (0–3), with higher total scores indicating greater anxiety severity. The Turkish validity and reliability study was conducted by Ulusoy et al. Cronbach’s α = 0.85 [32].

### 2.4. Sample Size and Sensitivity

Sample size was originally estimated with G*Power (version 3.1.9.7; Heinrich Heine University Düsseldorf, Germany) for an F test for repeated-measures ANOVA with a within–between interaction, assuming a medium effect (f = 0.25), α = 0.05 and power = 0.95. Because the achieved sample is fixed, we report a sensitivity analysis in place of a post hoc power calculation. With 40 and 39 participants per arm and α = 0.05, the design has 80% power to detect a differential effect of d = 0.64 and 90% power to detect d = 0.74. Effects smaller than this cannot be reliably detected. This value was therefore used as a design-specific sensitivity bound for the exploratory equivalence analysis; it should not be interpreted as a clinically validated equivalence margin [29].

### 2.5. Statistical Analysis

Descriptive statistics are presented as mean ± standard deviation for continuous variables and as frequency and percentage for categorical variables. Normality was evaluated with the Shapiro–Wilk test. Shapiro–Wilk rejected normality for at least one group or time point on six of the seven outcomes, and for the change scores in the control arm on six of seven; the analysis plan below was therefore designed to be robust to this.

Baseline characteristics were compared with independent-samples *t* tests and chi-square or Fisher’s exact tests as appropriate. Because several cell counts were small, Fisher’s exact test is reported for all 2 × 2 comparisons. Between-group differences on the psychological measures are reported as Cohen’s d with 95% confidence intervals alongside the significance test, and Mann–Whitney U is reported as a distribution-free check.

Group-level change was evaluated in four ways. (i) Mixed-design repeated-measures ANOVA with time as the within-subject factor and group as the between-subject factor, as in the original analysis. (ii) Linear mixed-effects models with a random intercept per participant, which accommodate the unequal arm sizes and provide an interpretable estimate of the differential change with a confidence interval. (iii) Analysis of covariance on the follow-up score adjusting for the baseline score. (iv) Aligned Rank Transform factorial ANOVA, a distribution-free procedure for factorial designs, as a robustness check against the normality violations. The time × group interaction was the primary test throughout, and false discovery rate correction was applied across the seven interaction tests.

The heterogeneity, equivalence, Bayesian, and Reliable Change Index analyses were added post hoc as exploratory analyses to further characterize the pattern observed in the primary analyses.

As exploratory post hoc analyses, two additional inferential procedures were applied to further characterize the absence of a differential mean effect. Two one-sided equivalence tests (TOST) were conducted on the differential change with bounds set at d = ±0.64, the smallest effect the design can detect with 80% power [28,29]; a conventional bound of d = ±0.50 is reported alongside. This bound was used only as a design-specific sensitivity threshold and was not treated as a theoretically or clinically established smallest effect size of interest. Bayes factors were computed for the same contrast using a default Jeffreys–Zellner–Siow prior (r = 0.707), and are reported as BF_01_, the evidence for the null relative to the alternative.

Individual response was examined in three ways. First, the variance of the change scores was compared between arms using Levene’s, Fligner–Killeen’s and the variance-ratio F test; under the standard assumption that comparator-arm change variance represents within-person random variation over the same interval, a larger intervention-arm variance is preliminary evidence of response heterogeneity [23,25]. Second, three-month test–retest correlations were computed separately by arm and compared with Fisher’s z. Third, each participant’s change was converted to a Reliable Change Index, RCI = (X_2_ − X_1_)/S_diff, where S_diff = √(2·SEM^2^) and SEM = SD_baseline·√(1 − α) [26]. Participants with |RCI| ≥ 1.96 were classified as reliably improved or reliably deteriorated. For each outcome the reliability coefficient entered into S_diff was the internal consistency estimate obtained in the present sample: α = 0.74 for the SAAS and α = 0.85 for the Beck Anxiety Inventory. Subscale-specific reliability coefficients were not available for the IRDS; the primary RCI calculation therefore used the total-scale reliability of α = 0.86 for the IRDS total score and for all four IRDS subscales. Because this assumption is likely to be optimistic for individual subscales, the entire classification was repeated across assumed subscale reliabilities of α = 0.70, 0.75, 0.80 and 0.86, with the coefficients for the SAAS, the Beck Anxiety Inventory and the IRDS total score held at their observed values throughout. The complete sensitivity results are reported in Supplementary Tables S1 and S2. Change was classified in the adaptive direction for each scale: a reliable decrease was counted as improvement for the Beck Anxiety Inventory, the SAAS and IRDS Approval Dependence, and a reliable increase was counted as improvement for IRDS Empathy, Trust in Others and Emotional Awareness. Because the IRDS total score sums subscales of opposing valence, its counts are reported as reliable decreases and increases rather than as improvement and deterioration, and it was excluded from the participant-level pattern classification.

Analyses were conducted in Python 3.12 (pandas, statsmodels, pingouin, scipy); the full script is provided as Supplementary Material. Statistical significance was set at *p* < 0.05, two-tailed.

## 3. Results

### 3.1. Sociodemographic and Clinical Characteristics

The surgical and control groups did not differ on any sociodemographic characteristic assessed (Table 1). Age was comparable between groups (34.15 ± 12.44 vs. 31.46 ± 7.92 years; *t* test *p* = 0.257, Mann–Whitney U *p* = 0.687). Sex distribution did not differ (2 vs. 6 men; Fisher’s exact *p* = 0.154), nor did marital status (*p* = 0.474), education (*p* = 0.321), employment (*p* = 0.498), income (*p* = 0.630) or smoking (*p* = 0.261).

### 3.2. Baseline and Follow-Up Group Differences

**Figure 2:**
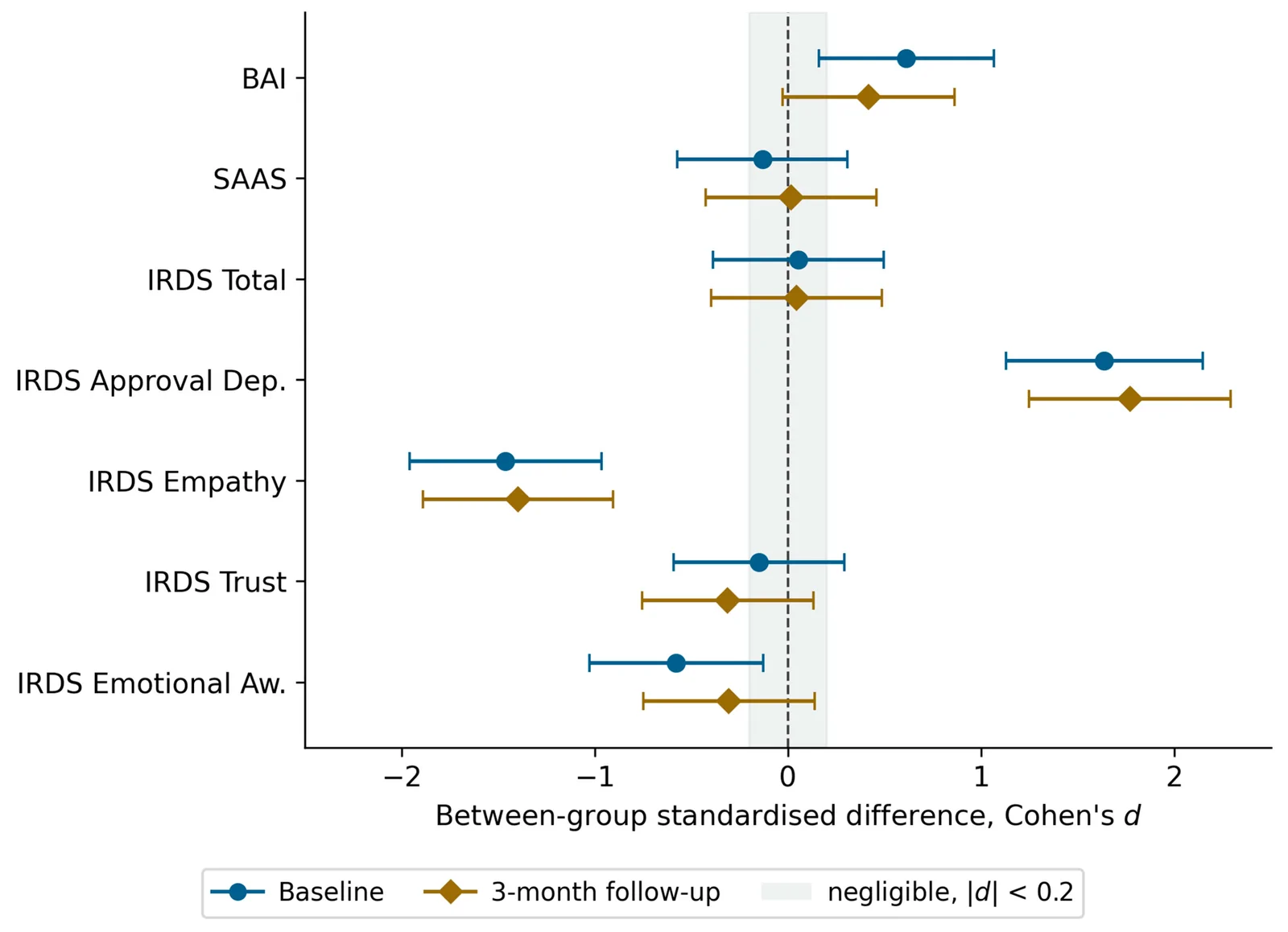
Standardised between-group differences on each outcome, at baseline (circles) and at three-month follow-up (diamonds), with 95% confidence intervals. Positive values indicate a higher score in the surgical group. The shaded band marks the conventional threshold for a negligible effect. Approval dependence, empathy and emotional awareness differ substantially between the arms before surgery, and each difference is essentially unchanged three months afterwards, indicating characteristics of the person rather than consequences of the procedure.

At baseline the surgical group reported significantly higher anxiety than controls (19.60 ± 5.55 vs. 16.62 ± 4.06; *p* = 0.008, d = 0.61) and a distinct interpersonal profile. Approval dependence was markedly higher in the surgical group (49.35 ± 8.97 vs. 35.00 ± 8.56; *p* < 0.001, d = 1.64), while empathy (21.10 ± 5.82 vs. 29.41 ± 5.54; *p* < 0.001, d = −1.46) and emotional awareness (35.92 ± 8.37 vs. 40.10 ± 5.82; *p* = 0.012, d = −0.58) were lower. Social appearance anxiety (*p* = 0.556), trust in others (*p* = 0.506) and IRDS total score (*p* = 0.816) did not differ. The approval dependence and empathy differences are very large by conventional standards and are the dominant feature of the baseline picture (Table 2, Figure 2).

### 3.3. Group-Level Change: Four Analytic Frameworks Agree

Mixed-design repeated-measures ANOVA showed no significant time × group interaction for any outcome (all *p* ≥ 0.196; all FDR-adjusted *p* ≥ 0.676). Linear mixed-effects models reproduced this pattern, with every interaction confidence interval comfortably spanning zero, and the Aligned Rank Transform ANOVA—which makes no distributional assumption—agreed on all seven outcomes (all *p* ≥ 0.335). Adjustment for age, sex, education, marital status, employment, income and smoking left the interaction estimates numerically unchanged, as it must: a time-invariant covariate cannot alter a within-subject interaction estimate in a two-occasion design. Significant group main effects persisted for anxiety, approval dependence, empathy and emotional awareness, reflecting the stable baseline differences rather than any differential change (Table 3).

### 3.4. What the Null Result Can and Cannot Exclude

**Figure 3:**
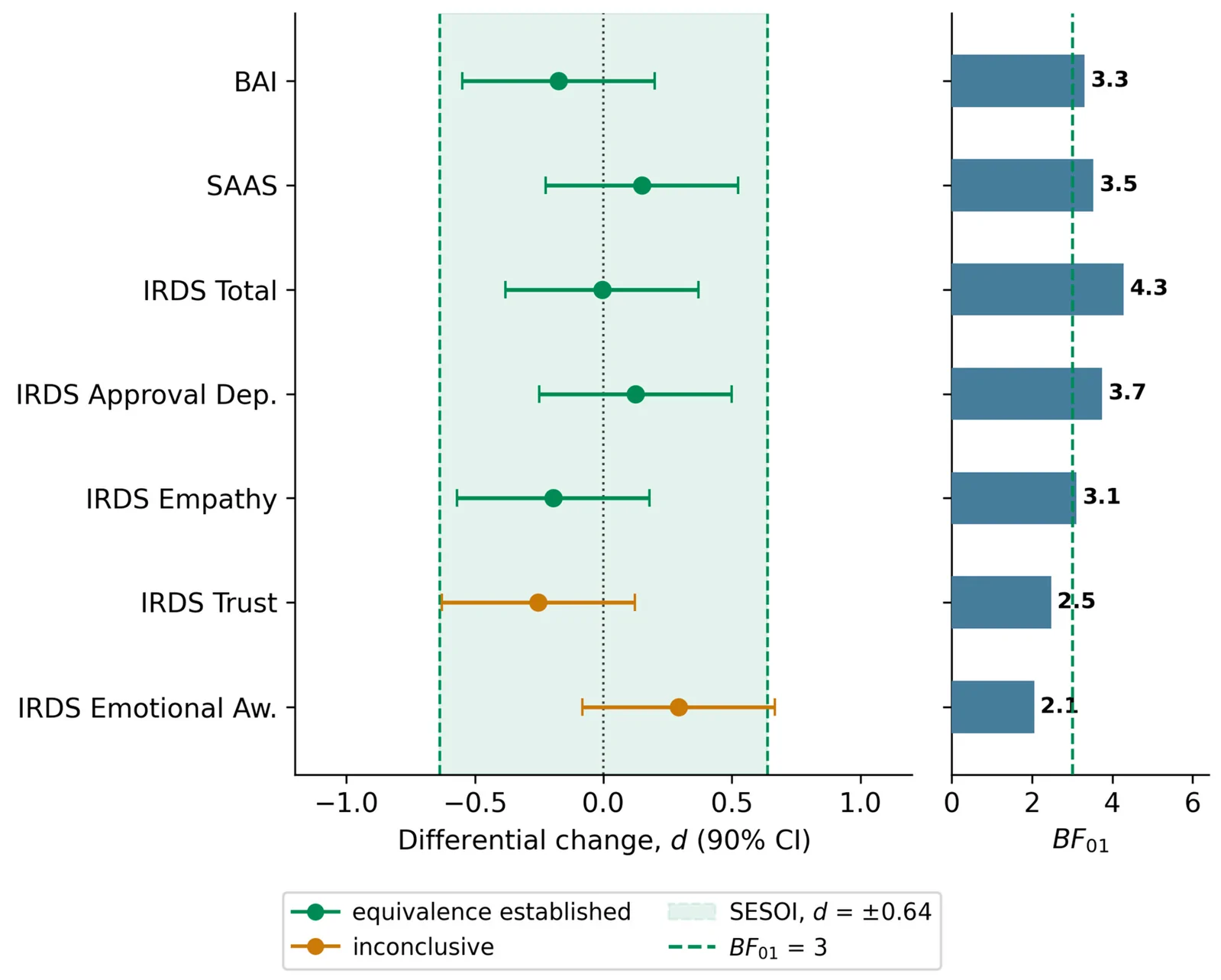
Equivalence and Bayesian evidence for the differential change. Left panel: the standardised differential change for each outcome with its 90% confidence interval, plotted against a design-specific sensitivity bound derived from the achieved sample size (dashed lines and shaded band, d = ±0.64). An interval lying entirely inside the band establishes statistical equivalence; green markers denote the five outcomes that do so after false discovery rate correction, amber markers the two that remain inconclusive. Right panel: Bayes factors expressing evidence for the null relative to the alternative. The dashed line marks BF01 = 3, the conventional threshold for moderate evidence; no outcome reaches strong evidence for the null.

Equivalence testing against the power-justified bound of d = ±0.64 established statistical equivalence for five of the seven outcomes after FDR correction: anxiety, social appearance anxiety, IRDS total, approval dependence and empathy (all FDR *p* ≤ 0.037). Trust in others (FDR *p* = 0.053) and emotional awareness (FDR *p* = 0.065) did not reach equivalence, meaning that for these two outcomes the data can exclude neither a meaningful differential effect nor its absence. Against the conventional bound of d = ±0.50, no outcome achieved equivalence after correction. Bayes factors were consistent with this reading: BF_01_ ranged from 2.05 to 4.28, indicating anecdotal to moderate—but never strong—support for the null (Table 4, Figure 3).

### 3.5. Individual Response Heterogeneity

**Figure 4:**
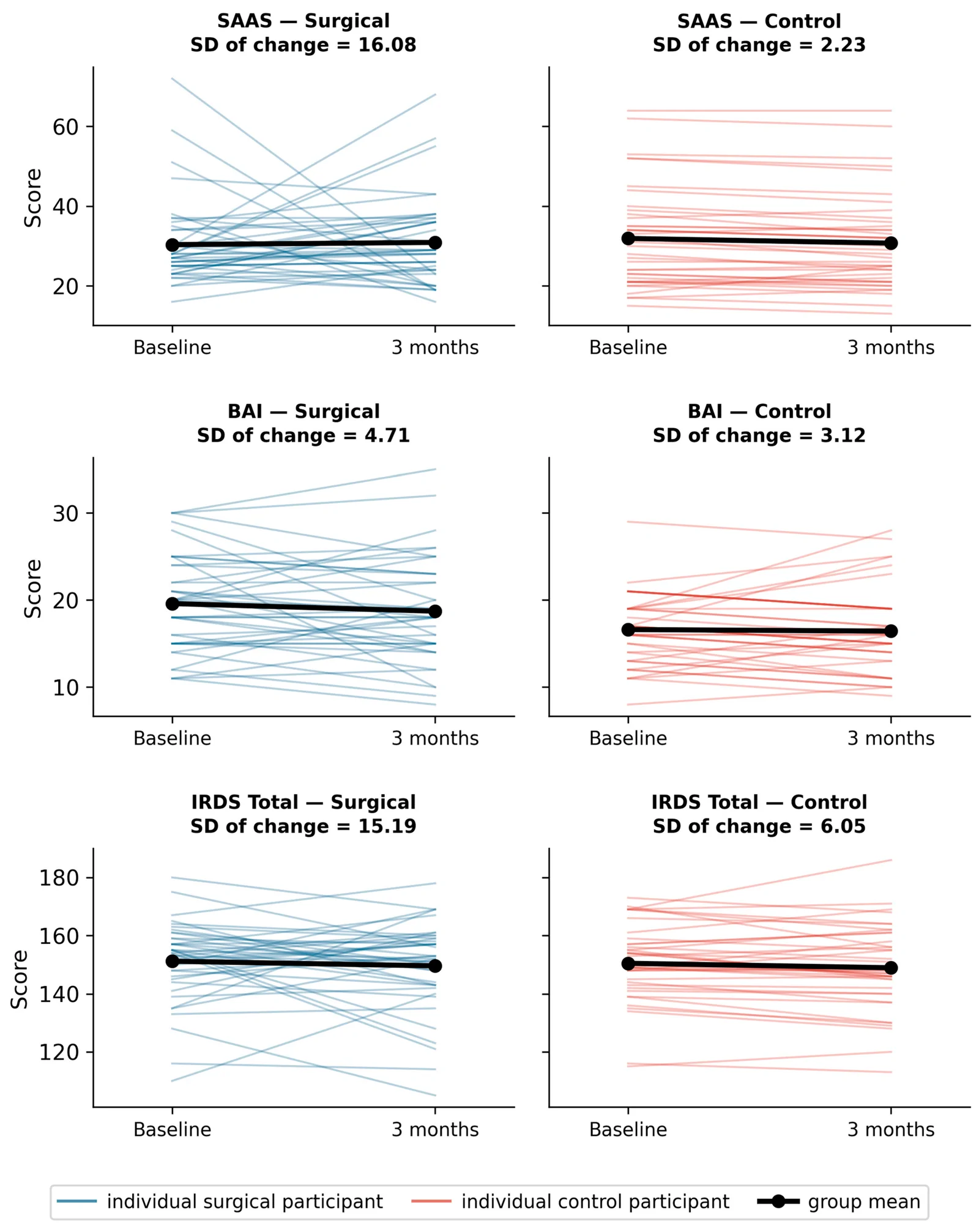
Individual trajectories from baseline to three months for three representative outcomes, by group. Each thin line is one participant; the heavy black line is the group mean. The standard deviation of individual change is given in each panel heading. The surgical mean is nearly flat in every panel, yet the individual lines fan out steeply in both directions, whereas control trajectories remain tightly bundled around their mean. This contrast illustrates that a near-flat group mean can coexist with substantially greater dispersion of individual change in the surgical group. Given the observational design, however, this pattern should be interpreted as compatible with, rather than definitive evidence of, heterogeneous individual responses to surgery.

**Figure 5:**
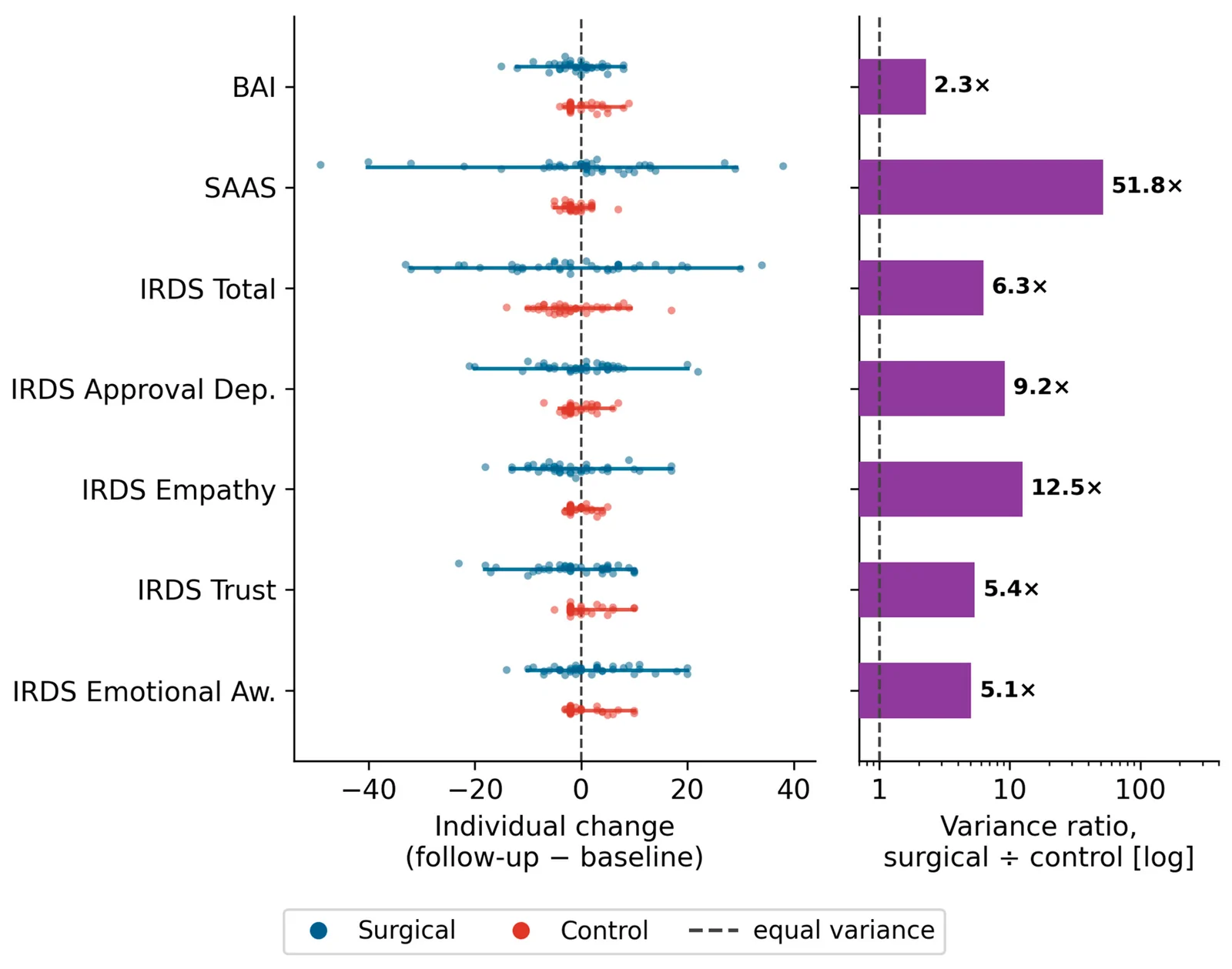
Dispersion of individual change. Left panel: every participant’s raw change score, with the horizontal bar marking the central 95% of each distribution. Right panel: the ratio of the surgical to the control change variance on a logarithmic axis; the dashed line marks equal variance. Values above one indicate that individual response was more dispersed in the surgical arm, which under the standard assumption that comparator-arm change variance represents within-person random variation is preliminary evidence of treatment response heterogeneity.

Although mean change did not differ, the dispersion of change differed dramatically. The variance of change scores was larger in the surgical arm on every outcome, by factors ranging from 2.3 (anxiety) to 51.8 (social appearance anxiety); all seven comparisons remained significant after FDR correction under Levene’s test (FDR *p* ≤ 0.045) and Fligner–Killeen’s test. On social appearance anxiety, control participants changed by between −5 and +7 points over three months, whereas surgical participants changed by between −49 and +38 (Table 5, Figure 4 and Figure 5).

**Figure 6:**
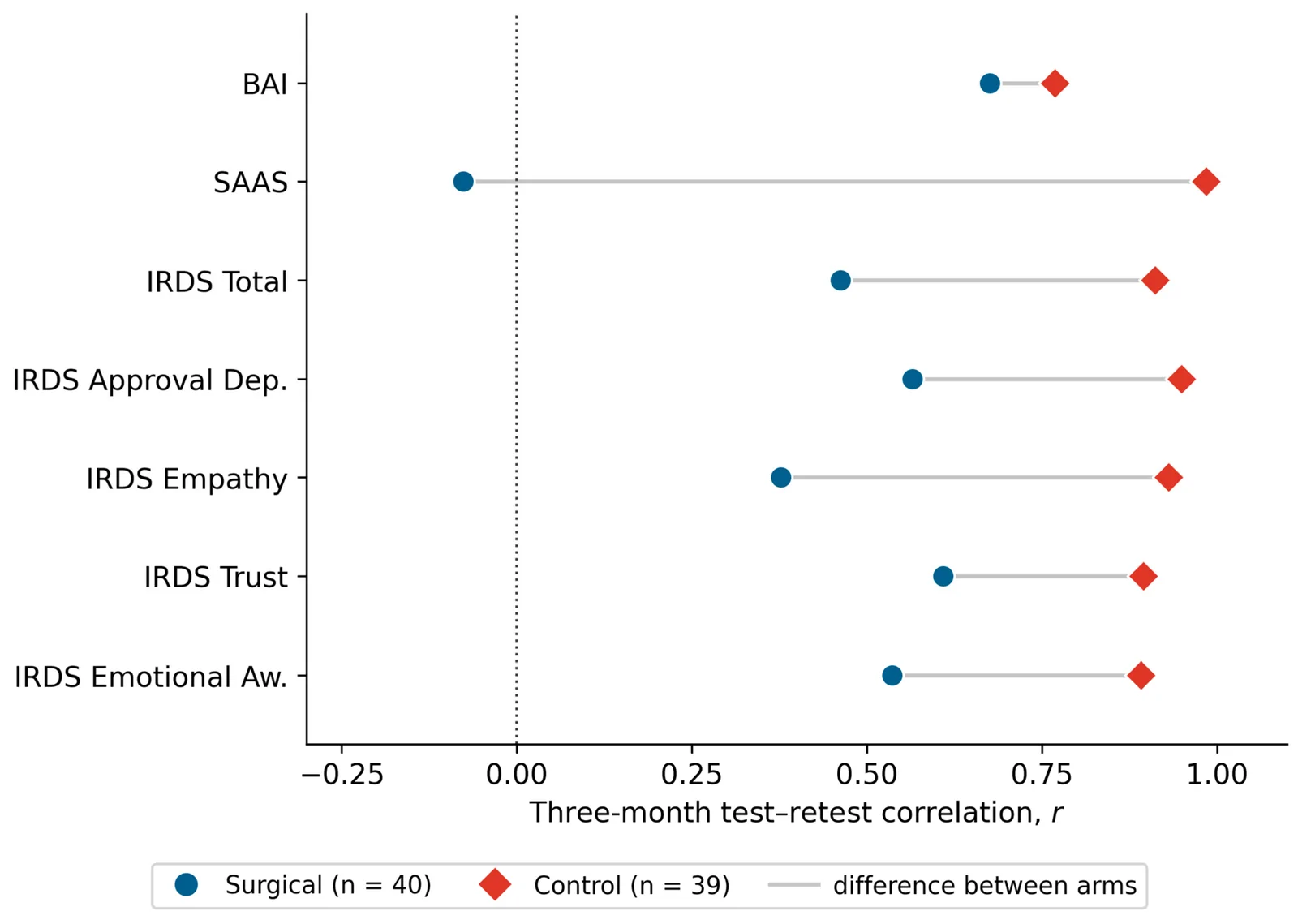
Three-month test–retest correlations for each outcome, by group, with the grey connector showing the gap between arms. Control correlations are uniformly high, reproducing the trait-like stability proposed in the previous version of this manuscript. Surgical correlations are substantially lower on six of seven outcomes, and for social appearance anxiety the correlation is effectively zero, meaning that a surgical patient’s score three months after surgery is statistically unrelated to the score recorded before it.

Three-month temporal stability showed the same asymmetry. In the control arm, test–retest correlations were uniformly high (r = 0.77 to 0.98), consistent with the trait-like stability the original manuscript proposed. In the surgical arm, they were substantially lower on six of seven outcomes, and for social appearance anxiety the correlation was effectively zero (r = −0.08, 95% CI [−0.38, 0.24]), meaning that a surgical patient’s appearance anxiety three months after surgery is statistically unrelated to what it was before. Fisher’s z tests confirmed the difference in stability on six of seven outcomes (all FDR *p* ≤ 0.002); only anxiety was comparable across arms (Table 5, Figure 6).

### 3.6. Reliable and Clinically Meaningful Change

**Figure 7:**
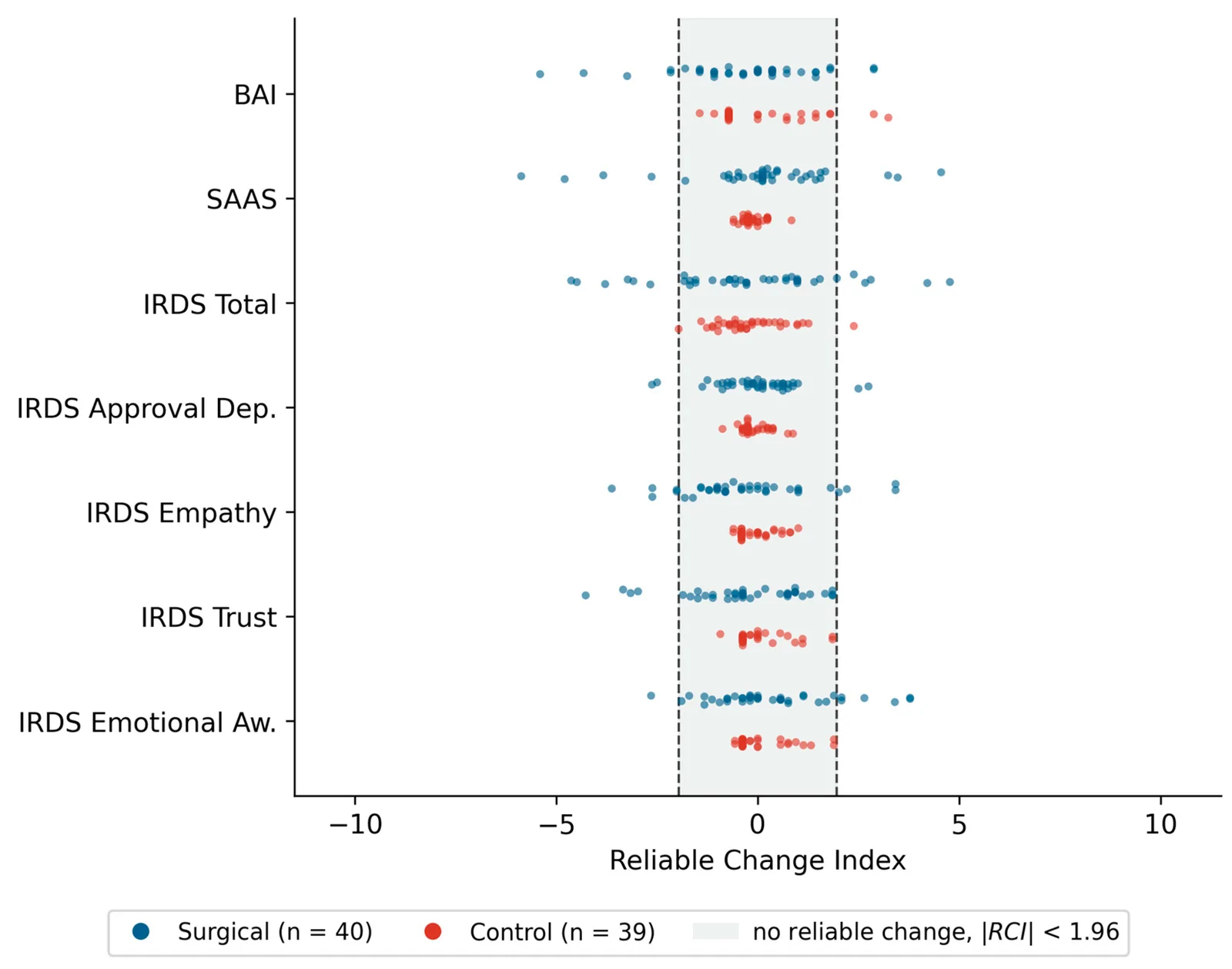
Reliable Change Index for every participant on every outcome. Each point is one participant; the shaded band spans |RCI| < 1.96, within which a change is no larger than measurement error alone would produce. Points to the left of the band represent reliable change in one direction and points to the right reliable change in the other. Surgical points lie outside the band far more often than control points, and they do so on both sides.

**Figure 8:**
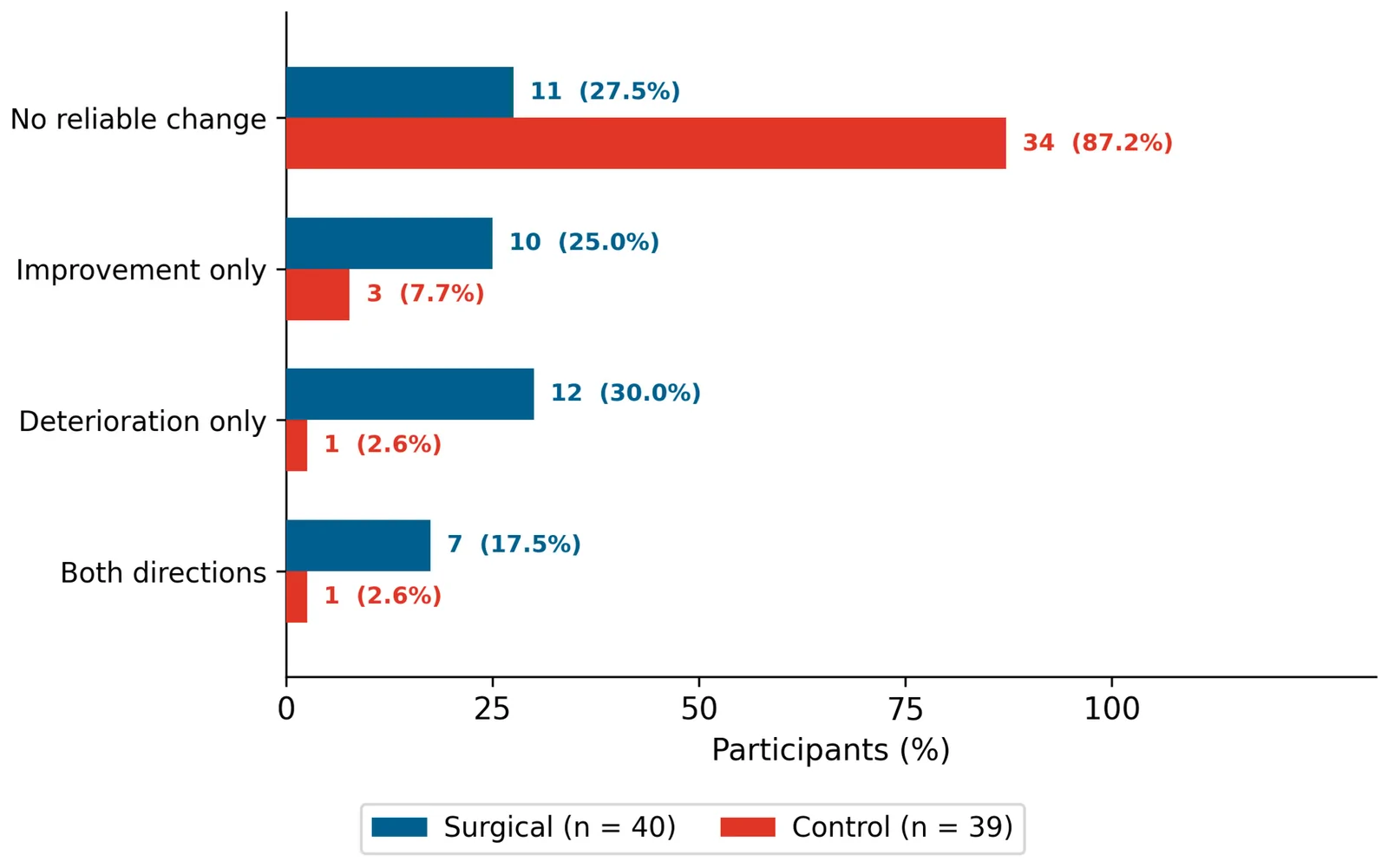
Classification of participants by their overall pattern of reliable change across the six individual outcomes (the IRDS total score is excluded because it sums subscales of opposing valence).

On the IRDS total score, six surgical patients showed a reliable decrease and six a reliable increase, against one and one among controls. On emotional awareness, nine improved and three deteriorated; on trust in others, four improved and eight deteriorated; on empathy, five improved and seven deteriorated (Table 6, Figure 7 and Figure 8).

At the level of the individual patient, 29 of 40 surgical participants (72.5%) showed reliable change on at least one outcome, compared with 5 of 39 controls (12.8%; Fisher’s exact *p* < 0.001). Within the surgical arm, 10 patients (25.0%) showed reliable improvement only, 12 (30.0%) reliable deterioration only, and 7 (17.5%) reliable change in both directions on different outcomes. The near balance between RCI-defined improvement and deterioration illustrates how changes in opposite directions may contribute to the near-zero mean change observed at the group level. A sensitivity analysis varying the assumed subscale reliability across α = 0.70, 0.75, 0.80 and 0.86 changed the absolute counts but not the pattern. The total number of reliable changes ranged from 45 to 66 in the surgical arm against 4 to 8 in the control arm, and the proportion of surgical participants showing reliable change on at least one outcome ranged from 55.0% to 72.5% against 5.1% to 12.8% of controls; the group difference remained significant at every level (all *p* < 0.001). As expected, lower assumed reliability widens the interval within which change is attributed to measurement error and therefore yields conservative counts, whereas the direction and the between-group contrast were unaffected. The complete per-outcome and participant-level results at each reliability level are given in Supplementary Tables S1 and S2.

## 4. Discussion

This prospective controlled study produced two findings that appear contradictory only until the level of analysis is made explicit. At the level of group means, aesthetic surgery was followed by no detectable differential change on any of seven psychological outcomes, a result that held under four analytic frameworks and survived correction for multiple testing. At the level of the individual patient, the surgical arm showed change that was several times more dispersed than the control arm, markedly lower temporal stability, and RCI-defined change in 72.5% of surgical participants compared with 12.8% of comparison participants. Both findings are correct. Together, these findings are compatible with greater heterogeneity of psychological trajectories in the surgical group, which may not be apparent from group-mean analyses alone. However, given the observational design, baseline group differences, and the inability to fully separate true response heterogeneity from measurement variability and other unmeasured factors, these findings should not be interpreted as demonstrating surgery-induced psychological change.

### 4.1. Baseline Differences and Who Seeks Surgery

Surgical candidates reported higher anxiety than controls, consistent with earlier work suggesting that individuals considering aesthetic surgery may present with heightened emotional distress or anticipatory concern [10]. Notably, they did not report higher social appearance anxiety, which weighs against the assumption that appearance-specific concern is the dominant motivator and suggests instead a broader emotional vulnerability. Notably, social appearance anxiety did not differ significantly between groups. Because motivations for seeking aesthetic surgery were not directly assessed, this finding should not be interpreted as evidence for or against any particular motivational pathway. The interpersonal differences were larger still. Approval dependence was more than a standard deviation and a half higher in the surgical group, and empathy nearly a standard deviation and a half lower—among the largest between-group differences reported in this literature. Interpreted through Social Comparison Theory [33], elevated approval dependence describes a disposition to anchor self-evaluation in external validation, which is precisely the disposition that renders discrepancies from idealised appearance standards personally consequential. These differences were present before surgery and were essentially unchanged three months afterwards, indicating characteristics of the person rather than consequences of the procedure.

### 4.2. Why the Group Means Are Flat

The absence of a mean effect replicates the more cautious strand of the existing literature [20,34,35] and stands against reports of postoperative gains in body satisfaction and self-esteem [4,36]. The equivalence analysis allows this to be stated more precisely than before: differential effects larger than d = 0.64 can be excluded for five of the seven outcomes, so the study does rule out large average effects. It does not rule out moderate ones. No outcome achieved equivalence at d = 0.50, and Bayes factors never exceeded 4.3. A study of this size can say that aesthetic surgery does not transform the average patient psychologically within three months; it cannot say that the average effect is nil.

The individual-level results provide one possible explanation for the near-zero mean differential change, namely that changes in opposite directions may offset one another at the group level. RCI-defined improvement only and deterioration only occurred in broadly comparable proportions in the surgical arm (25.0% and 30.0%, respectively), while 17.5% showed reliable change in both directions across different outcomes. This pattern illustrates how changes in opposing directions may contribute to the near-zero mean differential change at the group level. This is not merely a statistical curiosity but may reflect clinically meaningful individual variability: aesthetic surgery was associated with a near-zero average change but marked heterogeneity in individual psychological trajectories. The collapse in temporal stability points the same way. Control participants’ scores three months apart correlated between 0.77 and 0.98, reproducing the trait-like stability the original version of this manuscript proposed as an explanation for the null finding. In the surgical arm, the same correlations fell to between −0.08 and 0.68. These findings suggest that trait-like stability alone may not fully account for the observed pattern. The constructs showed high three-month stability in the comparison group but substantially lower stability in the surgical group. However, lower test–retest correlations may reflect genuine heterogeneous change, greater measurement variability, or other unmeasured differences between the groups, and therefore cannot be attributed specifically to surgery. These findings are consistent with greater instability in these dimensions among surgical participants, although the observational design does not permit attribution of this difference to surgery itself.

### 4.3. Clinical Implications

The clinical reading of a null result and of a heterogeneity result differ sharply. If surgery leaves psychological functioning untouched on average, then preoperative psychological assessment is a formality with little to detect. If approximately one quarter of participants show reliable improvement only and nearly one third show reliable deterioration only, with additional participants showing change in both directions across different outcomes, then identifying characteristics associated with these different trajectories may be clinically relevant if this pattern is confirmed in larger studies. If the observed pattern of individual variability is confirmed in larger and methodologically stronger studies, then identifying characteristics associated with more favorable or unfavorable psychological trajectories may become relevant to preoperative assessment. At present, however, these findings should be regarded as hypothesis-generating and do not establish clinically validated risk groups or justify causal attribution to surgery itself.

The direction of the present findings is consistent with a literature identifying who is likely to fare poorly. Reviews converge on body dysmorphic disorder, unrealistic expectations, younger age, male sex, minimal objective deformity and a history of mood or anxiety disorder as markers of poor psychosocial outcome [14,21], and body dysmorphic disorder is substantially more prevalent in cosmetic and plastic surgery settings than in the general population [22]. The present study excluded diagnosed body dysmorphic disorder at entry, so the deterioration observed here occurred in a cohort already screened for the single best-established risk marker. That is a clinically important observation: screening for body dysmorphic disorder alone does not appear to be sufficient. Although the present study was not powered to establish procedure-specific predictors, higher anxiety and approval dependence, together with lower empathy and emotional awareness, may represent candidate psychological domains for future investigation. Larger, adequately powered studies stratified by surgical procedure are needed to determine whether these characteristics identify patients at greater risk of adverse psychological outcomes.

Two further practical points follow. First, Emotional Awareness was the outcome on which improvement most clearly outnumbered deterioration (nine versus three), whereas Trust in Others showed the opposite pattern, with deterioration outnumbering improvement (eight versus four). Second, because reliable change was frequently confined to one or two dimensions within a patient rather than being global, follow-up that relies on a single global satisfaction item is unlikely to detect it.

### 4.4. Methodological Implications for the Field

If the pattern observed here generalises, it has consequences for how this literature should be read. A field that evaluates aesthetic surgery predominantly through group means may overlook substantial variability in individual psychological trajectories even when average change is close to zero. Reporting the dispersion of change alongside the mean, together with appropriately cautious individual-level analyses, may therefore provide a more complete description of postoperative psychological outcomes. The systematic review by Garbett et al. (2025) documents both the heterogeneity of this evidence base and the difficulty of drawing conclusions from it; part of that difficulty may be analytic rather than substantive [20]. Reporting the variance of change alongside the mean, and classifying individual change against a reliability criterion, costs nothing in additional data collection and can be added to any pre–post design that includes a comparator arm.

Several limitations qualify these findings. The three-month follow-up may not capture delayed change, and the results should be read as short-term. Generalizability is also limited by the small sample size and the marked predominance of women in the surgical group (38 of 40 participants). The findings therefore primarily describe short-term psychological trajectories in this specific, predominantly female mixed-procedure cohort and should not be generalized to aesthetic surgery populations as a whole. The sample, although adequate by the original power calculation, is small enough that only differential effects of d ≥ 0.64 are detectable, and the use of self-report measures may have introduced response bias. An additional limitation is the clinical heterogeneity of the surgical cohort. Abdominoplasty, breast surgery, liposuction, Brazilian butt lift, and rhinoplasty differ considerably in indication, patient expectations, recovery burden, visibility of the surgical result, and psychosocial meaning. The number of participants within each procedure category was insufficient for adequately powered procedure-specific analyses. Therefore, the present findings should be interpreted as describing psychological response heterogeneity across a mixed aesthetic-surgery cohort rather than as evidence that individual procedures have comparable psychological effects. Indeed, part of the observed response heterogeneity may reflect differences between procedure types. Larger prospective studies stratified by surgical procedure are required to determine whether particular operations are associated with distinct psychological trajectories. Body image satisfaction, self-esteem, depressive symptoms, satisfaction with the surgical result and quality of life were not assessed.

Three limitations bear specifically on the new analyses. First, the variance-comparison approach to response heterogeneity has known weaknesses. It assumes that comparator-arm change variance represents within-person random variation over the same interval, and because the correlation between an individual’s potential outcomes under treatment and no treatment is not identifiable from a parallel-group design, a variance difference is suggestive rather than conclusive evidence of true person-by-treatment interaction [34,35]. Definitive attribution would require a replicate crossover or n-of-1 design, which is not feasible for surgery. Although some variance differences were large in magnitude, the present design cannot determine how much of this variability reflects true individual response heterogeneity versus measurement variability, regression to the mean, baseline heterogeneity, procedure-related differences, or other unmeasured factors.

Second, a lower test–retest correlation in the surgical arm is consistent both with genuine heterogeneous change and with greater measurement noise following surgery; the present design cannot fully separate these. Third, the Reliable Change Index depends on the reliability coefficient assumed, and subscale-specific alphas were not available for the IRDS subscales, so a total-scale value was used with a sensitivity analysis across plausible alternatives. Item-level data should be retained in future work so that subscale reliabilities can be estimated directly.

Finally, this was an observational study. Participants were not randomised to surgery, and the two arms differed substantially at baseline on approval dependence, empathy and anxiety. The differential change results are therefore not causal estimates of a surgical effect; they describe what happened to two non-equivalent groups over the same interval. Analysis of covariance on the follow-up score adjusting for baseline returned a significant group difference for approval dependence (*p* = 0.007), but with groups this unbalanced at baseline, that analysis is subject to Lord’s Paradox and is not interpretable as differential change; it is reported for completeness only.

## 5. Conclusions

Surgical candidates presented with higher anxiety and a distinct interpersonal profile, and these differences persisted unchanged across the follow-up period. No differential change in group means was detected under any of four analytic frameworks, and equivalence testing excluded differential effects larger than d = 0.64 on five of seven outcomes—though moderate effects could not be excluded on any. Beneath that flat average, however, individual change in the surgical group was several times more dispersed than in controls, three-month stability was substantially lower, and 72.5% of surgical participants showed RCI-defined change on at least one outcome, compared with 12.8% of comparison participants, with improvement-only and deterioration-only classifications occurring in broadly comparable proportions. These exploratory individual-level findings are compatible with greater heterogeneity of psychological trajectories in the surgical group, but they cannot establish that the observed variability was caused by aesthetic surgery itself.

The present findings suggest little average differential change but substantial variability in short-term individual psychological trajectories within this predominantly female, mixed-procedure surgical sample, consistent with possible response heterogeneity. If confirmed in larger, better-matched prospective studies, then this pattern may support efforts to identify patient characteristics associated with more favorable or unfavorable psychological trajectories after aesthetic surgery. For now, the present findings should be regarded as hypothesis-generating rather than as evidence of clinically established surgery-related response heterogeneity. Future studies should report the dispersion of change alongside its mean, classify individual change against a reliability criterion, follow patients beyond three months, and recruit samples large enough to model who improves and who does not.

## Figures and Tables

**Table 1:** Sociodemographic characteristics by group. p values from independent-samples *t* test (age) and Fisher’s exact test (categorical variables). Body mass index, reported in the previous version, is not available in the analysis dataset and has been removed.

Variable	Surgical (*n* = 40)	Control (*n* = 39)	*p*
Age (years), M ± SD	34.15 ± 12.44	31.46 ± 7.92	0.257
Sex, *n* (%)			0.154
Male	2 (5.0)	6 (15.4)	
Female	38 (95.0)	33 (84.6)	
Marital status, *n* (%)			0.474
Single	25 (62.5)	28 (71.8)	
Married	15 (37.5)	11 (28.2)	
Education, *n* (%)			0.321
Primary	11 (27.5)	12 (30.8)	
High school	16 (40.0)	20 (51.3)	
University	13 (32.5)	7 (17.9)	
Employed, *n* (%)	19 (47.5)	15 (38.5)	0.498
High income, *n* (%)	14 (35.0)	11 (28.2)	0.630
Current smoker, *n* (%)	15 (37.5)	20 (51.3)	0.261

**Table 2:** Baseline comparison of psychological measures. MWU = Mann–Whitney U. Positive d indicates a higher score in the surgical group.

Outcome	Surgical M ± SD	Control M ± SD	*p*	MWU *p*	d [95% CI]
Anxiety BAI	19.60 ± 5.55	16.62 ± 4.06	0.008	0.021	0.61 [0.16, 1.07]
SAAS	30.35 ± 10.79	31.90 ± 12.42	0.556	0.735	−0.13 [−0.58, 0.31]
IRDS Total	151.22 ± 13.88	150.51 ± 13.21	0.816	0.556	0.05 [−0.39, 0.50]
IRDS Approval Dependence	49.35 ± 8.97	35.00 ± 8.56	<0.001	<0.001	1.64 [1.12, 2.15]
IRDS Empathy	21.10 ± 5.82	29.41 ± 5.54	<0.001	<0.001	−1.46 [−1.96, −0.96]
IRDS Trust in Others	44.85 ± 7.84	46.00 ± 7.43	0.506	0.527	−0.15 [−0.59, 0.29]
IRDS Emotional Awareness	35.92 ± 8.37	40.10 ± 5.82	0.012	0.044	−0.58 [−1.03, −0.13]

**Table 3:** Time × group interaction across four analytic frameworks. RM-ANOVA = mixed-design repeated-measures analysis of variance; LMM = linear mixed-effects model with a random intercept, b is the interaction coefficient in scale points; ART = Aligned Rank Transform ANOVA. FDR correction is applied across the seven RM-ANOVA interaction tests.

Outcome	RM-ANOVA F(1, 77)	*p*	ηp^2^	LMM b [95% CI]	LMM *p*	ART *p*	FDR *p*
Anxiety	0.596	0.443	0.008	−0.70 [−2.46, 1.07]	0.440	0.806	0.676
SAAS	0.443	0.508	0.006	1.73 [−3.36, 6.82]	0.506	0.629	0.676
IRDS Total	0.001	0.981	0.000	−0.06 [−5.19, 5.06]	0.981	0.884	0.981
IRDS Approval Dep.	0.310	0.579	0.004	0.77 [−1.95, 3.49]	0.578	0.574	0.676
IRDS Empathy	0.745	0.391	0.010	−1.09 [−3.55, 1.38]	0.388	0.335	0.676
IRDS Trust	1.262	0.265	0.016	−1.55 [−4.25, 1.15]	0.261	0.623	0.676
IRDS Emotional Aw.	1.700	0.196	0.022	1.80 [−0.91, 4.51]	0.192	0.437	0.676

**Table 4:** Equivalence tests and Bayesian evidence for the differential change. TOST = two one-sided tests. A design-specific sensitivity bound derived from the achieved sample size, d = 0.64, is the smallest differential effect the design can detect with 80% power. BF_01_ > 3 is conventionally read as moderate evidence for the null.

Outcome	Differential Change d [90% CI]	TOST *p* (d = ±0.64)	FDR *p*	Equivalent?	TOST *p* (d = ±0.50)	BF_01_
Anxiety	−0.17 [−0.55, 0.20]	0.021	0.037	Yes	0.076	3.30
SAAS	0.15 [−0.22, 0.52]	0.017	0.037	Yes	0.062	3.53
IRDS Total	−0.01 [−0.38, 0.37]	0.003	0.022	Yes	0.016	4.28
IRDS Approval Dep.	0.13 [−0.25, 0.50]	0.013	0.037	Yes	0.050	3.74
IRDS Empathy	−0.19 [−0.57, 0.18]	0.026	0.037	Yes	0.089	3.10
IRDS Trust	−0.25 [−0.63, 0.12]	0.046	0.053	No	0.138	2.48
IRDS Emotional Aw.	0.29 [−0.08, 0.67]	0.065	0.065	No	0.181	2.05

**Table 5:** Dispersion of individual change and three-month temporal stability by group. The variance ratio is the surgical change variance divided by the control change variance.

Outcome	SD of Change, Surgical	SD of Change, Control	Variance Ratio	Levene FDR *p*	Stability r, Surgical	Stability r, Control	Fisher FDR *p*
Anxiety	4.71	3.12	2.3×	0.045	0.68	0.77	0.404
SAAS	16.08	2.23	51.8×	<0.001	−0.08	0.98	<0.001
IRDS Total	15.19	6.05	6.3×	<0.001	0.46	0.91	<0.001
IRDS Approval Dep.	8.23	2.72	9.2×	<0.001	0.56	0.95	<0.001
IRDS Empathy	7.57	2.14	12.5×	<0.001	0.38	0.93	<0.001
IRDS Trust	7.91	3.41	5.4×	<0.001	0.61	0.90	0.002
IRDS Emotional Aw.	7.89	3.51	5.1×	<0.001	0.54	0.89	0.001

**Table 6:** Reliable Change Index classification by outcome and group, computed with α = 0.86 for the IRDS total score and subscales, α = 0.85 for the Beck Anxiety Inventory and α = 0.74 for the SAAS. Improvement denotes a reliable change in the adaptive direction for that scale: a decrease for the Beck Anxiety Inventory, the SAAS and Approval Dependence, and an increase for Empathy, Trust in Others and Emotional Awareness. For the IRDS total score, which sums subscales of opposing valence, the three figures are reliable decreases/no reliable change/reliable increases. Fisher’s exact test compares the proportion of participants showing any reliable change. Results across assumed subscale reliabilities of α = 0.70 to 0.86 are given in Supplementary Table S1.

Outcome	Surgical: Improved/Unchanged/Deteriorated	Control: Improved/Unchanged/Deteriorated	Fisher *p*	FDR *p*
Anxiety	5/33/2	0/37/2	0.154	0.154
SAAS	4/33/3	0/39/0	0.012	0.016
IRDS Total	6/28/6	1/37/1	0.006	0.011
IRDS Approval Dep.	2/36/2	0/39/0	0.116	0.135
IRDS Empathy	5/28/7	0/39/0	<0.001	0.001
IRDS Trust	4/28/8	2/37/0	0.006	0.011
IRDS Emotional Aw.	9/28/3	2/37/0	0.006	0.011

## Data Availability

The data supporting the findings of this study are available from the corresponding authors upon reasonable request. The dataset will also be provided to the journal as Supplementary Material during the submission process. Due to privacy and ethical considerations, the data are not deposited in a publicly accessible repository.

## Supplementary Materials

The following supporting information can be downloaded at: https://www.mdpi.com/article/10.3390/jcm15207982/s1, Table S1. Per-outcome RCI classification at four assumed subscale reliabilities. Table S2. Participant-level pattern of reliable change at four assumed subscale reliabilities.
