# Supplementary material for: Beyond the Group Mean: Individual Response Heterogeneity in Social Appearance Anxiety, Anxiety and Interpersonal Functioning Following Aesthetic Surgery—A Prospective Controlled Study
Source: J Clin Med. 2026 Oct 15;15(20):7982. doi: 10.3390/jcm15207982 (PMC13706551; doi:10.3390/jcm15207982)
Supplement: Supplementary file 1 [file jcm-15-07982-s001.zip › jcm-4529200-supplementary.pdf]

Supplementary Material

Reliable Change Index: sensitivity to the assumed reliability coefficient

Manuscript jcm-4529200 · Öcük & Kocamer Şahin · Journal of Clinical Medicine

The Reliable Change Index (RCI) requires a reliability coefficient for each measure, entering the calculation through the standard error of measurement, SEM = SD_baseline · √(1 − α), and the standard error of the difference, S_diff = √(2 · SEM²). For the SAAS and the Beck Anxiety Inventory the internal consistency estimates obtained in the present sample were used (α = .74 and α = .85, respectively). Subscale-specific reliability coefficients were not available for the Interpersonal Relationships Dimensions Scale, so the total-scale value of α = .86 was applied to the IRDS total score and to all four subscales in the primary analysis reported in Table 6 of the main text.

Because a total-scale coefficient is likely to overstate the reliability of individual subscales, and because a lower assumed reliability widens the band within which change is attributed to measurement error, the entire classification was repeated across assumed subscale reliabilities of α = .70, .75, .80 and .86. The coefficients for the SAAS, the Beck Anxiety Inventory and the IRDS total score were held at their observed values throughout, so the three rows carrying those measures are identical across the four panels of Table S1 by construction; only the four IRDS subscale rows vary.

Table S1. Per-outcome RCI classification at four assumed subscale reliabilities

Assumed subscale reliability α = .70

Outcome	α used	Surgical improved / unchanged / deteriorated	Control improved / unchanged / deteriorated	Fisher p	FDR p
Anxiety (BAI)	α = .85	5 / 33 / 2	0 / 37 / 2	.154	.154
SAAS	α = .74	4 / 33 / 3	0 / 39 / 0	.012	.041
IRDS Total †	α = .86	6 / 28 / 6	1 / 37 / 1	.006	.041
IRDS Approval Dependence	α = .70	2 / 36 / 2	0 / 39 / 0	.116	.135
IRDS Empathy	α = .70	3 / 34 / 3	0 / 39 / 0	.026	.060
IRDS Trust in Others	α = .70	0 / 36 / 4	0 / 39 / 0	.116	.135
IRDS Emotional Awareness	α = .70	4 / 35 / 1	0 / 39 / 0	.055	.096
**Total reliable changes**	—	**45**	**4**		

Assumed subscale reliability α = .75

Outcome	α used	Surgical improved / unchanged / deteriorated	Control improved / unchanged / deteriorated	Fisher p	FDR p
Anxiety (BAI)	α = .85	5 / 33 / 2	0 / 37 / 2	.154	.154
SAAS	α = .74	4 / 33 / 3	0 / 39 / 0	.012	.021
IRDS Total †	α = .86	6 / 28 / 6	1 / 37 / 1	.006	.021
IRDS Approval Dependence	α = .75	2 / 36 / 2	0 / 39 / 0	.116	.135
IRDS Empathy	α = .75	4 / 31 / 5	0 / 39 / 0	.002	.017
IRDS Trust in Others	α = .75	0 / 36 / 4	0 / 39 / 0	.116	.135
IRDS Emotional Awareness	α = .75	6 / 33 / 1	0 / 39 / 0	.012	.021
**Total reliable changes**	—	**50**	**4**		

Assumed subscale reliability α = .80

Outcome	α used	Surgical improved / unchanged / deteriorated	Control improved / unchanged / deteriorated	Fisher p	FDR p
Anxiety (BAI)	α = .85	5 / 33 / 2	0 / 37 / 2	.154	.154
SAAS	α = .74	4 / 33 / 3	0 / 39 / 0	.012	.027
IRDS Total †	α = .86	6 / 28 / 6	1 / 37 / 1	.006	.023
IRDS Approval Dependence	α = .80	2 / 36 / 2	0 / 39 / 0	.116	.135
IRDS Empathy	α = .80	5 / 29 / 6	0 / 39 / 0	< .001	.003
IRDS Trust in Others	α = .80	3 / 32 / 5	2 / 37 / 0	.087	.122

Outcome	α used	Surgical improved / unchanged / deteriorated	Control improved / unchanged / deteriorated	Fisher p	FDR p
IRDS Emotional Awareness	α = .80	7 / 31 / 2	2 / 37 / 0	.048	.083
**Total reliable changes**	—	**58**	**8**		

Assumed subscale reliability α = .86

Outcome	α used	Surgical improved / unchanged / deteriorated	Control improved / unchanged / deteriorated	Fisher p	FDR p
Anxiety (BAI)	α = .85	5 / 33 / 2	0 / 37 / 2	.154	.154
SAAS	α = .74	4 / 33 / 3	0 / 39 / 0	.012	.016
IRDS Total †	α = .86	6 / 28 / 6	1 / 37 / 1	.006	.011
IRDS Approval Dependence	α = .86	2 / 36 / 2	0 / 39 / 0	.116	.135
IRDS Empathy	α = .86	5 / 28 / 7	0 / 39 / 0	< .001	.001
IRDS Trust in Others	α = .86	4 / 28 / 8	2 / 37 / 0	.006	.011
IRDS Emotional Awareness	α = .86	9 / 28 / 3	2 / 37 / 0	.006	.011
**Total reliable changes**	—	**66**	**8**		

Improvement denotes a reliable change (|RCI| ≥ 1.96) in the adaptive direction for that scale: a decrease for the Beck Anxiety Inventory, the SAAS and IRDS Approval Dependence, and an increase for IRDS Empathy, Trust in Others and Emotional Awareness. † For the IRDS total score, which sums subscales of opposing valence, improvement is not defined and the three figures are reliable decreases / no reliable change / reliable increases. The α used column shows that the SAAS, Beck Anxiety Inventory and IRDS total score retain their own observed coefficients at every level, so only the four subscale rows vary across panels. Fisher's exact test compares the proportion of participants showing any reliable change; FDR correction is applied across the seven outcomes within each panel. Surgical n = 40, control n = 39.

Table S2. Participant-level pattern of reliable change at four assumed subscale reliabilities

α	Surgical none	improve only	deteriorate only	both	Control none	improve only	deteriorate only	both	Any change (%) surgical vs control	p
.70	18	10	8	4	37	0	2	0	55.0 vs 5.1	< .001
.75	17	10	8	5	37	0	2	0	57.5 vs 5.1	< .001
.80	16	9	8	7	34	3	1	1	60.0 vs 12.8	< .001
.86	11	10	12	7	34	3	1	1	72.5 vs 12.8	< .001

Each participant is classified by their pattern of reliable change across the six individual outcomes; the IRDS total score is excluded because it sums subscales of opposing valence and would double-count its constituent subscales. Percentages are of 40 surgical and 39 control participants. p values are from Fisher's exact test comparing the proportion showing any reliable change.

Interpretation

- The absolute number of reliable changes rises monotonically with the assumed reliability, from 45 to 66 in the surgical arm and from 4 to 8 in the control arm, which is the expected consequence of a narrower measurement-error band at higher α.
- The proportion of surgical participants showing reliable change on at least one outcome ranges from 55.0% to 72.5%, against 5.1% to 12.8% of controls. The between-group difference is significant at every reliability level (all p < .001), and the surgical-to-control ratio remains between 5.6 and 10.8 throughout.
- Reliable change occurs in both directions in the surgical arm at every reliability level. The improvement-only and deterioration-only counts stay close together across the whole range (10 vs 8 at α = .70, rising to 10 vs 12 at α = .86), so the observation that changes in opposing directions coexist does not depend on the reliability assumption.
- The choice of α is therefore consequential for the absolute counts and should be reported explicitly, but it does not alter the direction, the between-group contrast, or the bidirectionality that the analysis is used to illustrate. The primary analysis at α = .86 is the least conservative of the four and is reported as such in the main text.

Reproducibility

The RCI classification and this sensitivity analysis can be reproduced from the study dataset with the following procedure, implemented in Python 3.12 (pandas, numpy, scipy, statsmodels).

```
SEM       = SD_baseline * sqrt(1 - alpha)      # SD_baseline pooled across both arms
S_diff    = sqrt(2 * SEM ** 2)
RCI       = (score_followup - score_baseline) / S_diff
reliable  = abs(RCI) >= 1.96

# adaptive direction
#   decrease = improvement : Beck Anxiety Inventory, SAAS, IRDS Approval Dependence
#   increase = improvement : IRDS Empathy, Trust in Others, Emotional Awareness
#   IRDS total score       : reported as decrease / none / increase, not classified

alpha_fixed = {"BAI": .85, "SAAS": .74, "IRDS total": .86}
for alpha_subscale in (.70, .75, .80, .86):
    # subscale alpha varies; the three fixed coefficients above are held constant
    ...
```
